# Supplementary material for: Development and validation of a pharmaceutical assessment screening tool to prioritise patient care in a tertiary care hospital
Source: PLoS One. 2023 Mar 3;18(3):e0282342. doi: 10.1371/journal.pone.0282342 (PMC9983860; doi:10.1371/journal.pone.0282342)
Supplement: S1 File — (DOCX) [file pone.0282342.s001.docx]

**Supplementary files – Minimal data Set**

**Delphi Survey Round 1 Results – Expert Feedback**

**Total number of experts: 24 (Response rate: 24/24 = 100%)**

**Question 1: (H) High Alert Medications -** Patient requiring High Alert Medications (HAM).

Expert’s responses

| **Likert Scale** | **Relevance** | **Completeness** |
| --- | --- | --- |
| 1 | 0 (0%) | 0 (0%) |
| 2 | 0 (0%) | 1 (4.2%) |
| 3 | 3 (12.5%) | 6 (25%) |
| 4 | 4 (16.7%) | 8 (33.3%) |
| 5 | 17 (70.8%) | 9 (37.5%) |

**Consensus

|  | % | **Consensus achieved** |
| --- | --- | --- |
| Relevance | 87.5% | Yes |
| Completeness | 70.8% | No |

**Consensus is achieved if ≥75% of experts rate agree or strongly agree in the Likert scale

1. *(a) Does the criteria above require any modification and addition in terms of content?*

Total number of comments/ suggestions: 7

Suggestions/comments given by the experts:

| Expert A | 1 HAM drug = 1 point? or yes = 1 point |
| --- | --- |
| Expert B | Yes. A suggestion would be to narrow down the HAM list to those relevant for General Medical Wards. E.g. GAs, inotropes, NMB drugs etc won't be used in these wards. Under the current HAM list, oral hypoglycaemics (e.g. Metformin) is also listed. Being so, there is a high likelihood that this criteria will definitely be scored as +1 as there is a high likelihood patients admitted to general medical ward will have DM and thus be on some form of OHA and even insulin. Hence, perhaps it will be more meaningful to relook into this list and list down high risk medications or perhaps score this criteria when patient is on > 2 or 3 HAMs listed? |
| Expert C | Yes. How is the scoring works? Is it one score per HAM or one score regardless of the number of HAM the patient is on? |
| Expert D | Yes, would put significant weightage for cases requiring urgent pharmaceutical interventions |
| Expert E | May consider 'use more than 24 hours' |
| Expert F | Yes. Suggest to revise the list of HAM for the purpose of clinical pharmacy clerking. The existing list is more relevant to "HAMs which are kept in ward".   HAM may vary between disciplines and ward settings depending on the types of medicines used and patients treated. Some items in the list are not relevant for clerking, e.g. dialysis solution, radiocontrast agent etc. Furthermore, the list is incomplete if it is intended as a reference for clerking.   For example, only IV digoxin or amiodarone is in the list. However, patient with PO digoxin or amiodarone should be given attention in case follow up too. Biologics, DAPT (which antiplatelet is not in the list of antithrombotic), steroid (in tapering up/ down dose), diuretics are also HAMs in other settings too.   However, the HAM list will be too exhaustive if all HAM is included. Suggest separated HAM list according to each discipline and ward settings. For examples: - HAM in medical settings - HAM in acute care settings - HAM in surgical settings - HAM in long-term care settings (e.g. BEER, STOP, START criteria in geriatrics) |
| Expert G | Suggest to specify more clearly which types of HAM requires clerking; or maybe certain HAMs/more than one HAM drugs only will score 1..Not all HAM brings attention for clerking, e.g. if is on Injection KCL just for correction. It’s doesn't bring a necessity for clerking. |

1. *(b) Does the criteria above require any modification in terms of sentence structure?*

Total number of comments/ suggestions: 3

Suggestions/comments given by the experts:

| Expert A | Comment is mentioned above in 1.  Yes. A suggestion would be to narrow down the HAM list to those relevant for General Medical Wards. E.g GAs, inotropes, NMB drugs etc won't be used in these wards. Under the current HAM list, oral hypoglycaemic (e.g. Metformin) is also listed. Being so, there is a high likelihood that this criteria will definitely be scored as +1 as there is a high likelihood patients admitted to general medical ward will have DM and thus be on some form of OHA and even insulin. Hence, perhaps it will be more meaningful to relook into this list and list down high risk medications or perhaps score this criteria when patient is on > 2 or 3 HAMs listed? |
| --- | --- |
| Expert B | Suggestion: to add 'Requiring HAM upon admission',  Query: how about patient receiving HAM later after admission, does the score is updatable as long as patient was in the ward |
| Expert C | Modified sentence to:   Patient requiring High Alert Medications (HAM) which bear a heightened risk of causing significant patient harm when these medications are used in error. |

**Question 2: (O) Organ Dysfunction** - Presence of altered organ function of at least one organ in an acutely ill patient such that homeostasis cannot be maintained without intervention.

Expert’s responses

| **Likert Scale** | **Relevance** | **Completeness** |
| --- | --- | --- |
| 1 | 0 (0%) | 0 (0%) |
| 2 | 0 (0%) | 1 (4.2%) |
| 3 | 0 (0%) | 2 (8.3%) |
| 4 | 6 (25%) | 8 (33.3%) |
| 5 | 18 (75%) | 13 (54.2%) |

**Consensus

|  | % | **Consensus achieved** |
| --- | --- | --- |
| Relevance | 100% | Yes |
| Completeness | 87.5% | Yes |

**Consensus is achieved if ≥75% of experts rates agree or strongly agree in the Likert scale

1. *(a) Does the criteria above require any modification and addition in terms of content?*

Total number of comments/ suggestions: 4

Suggestions/comments given by the experts:

| Expert A | How to define acutely ill patient? |
| --- | --- |
| Expert B | Must the patient be in an acute state of illness to be qualified for this criteria? For e.g. if an U/L CKD patient is admitted for BSP monitoring and insulin optimisation, should this criteria (i.e. kidney) be taken into account since no 'intervention' is currently needed in terms of the patient's CKD? Or should the statement just state: Presence of altered organ function of at least one organ. |
| Expert C | Yes. The phrase “homeostasis cannot be maintained without intervention" is unclear. |
| Expert D | Yes, would weightage on need or urgent interventions only. |

1. *(b) Does the criteria above require any modification in terms of sentence structure?*

Total number of comments/ suggestions: 6

Suggestions/comments given by the experts:

| Expert A | Kindly refine above issue. |
| --- | --- |
| Expert B | Comment as mentioned above in 1.  Must the patient be in an acute state of illness to be qualified for this criteria? For e.g. if an U/L CKD patient is admitted for BSP monitoring and insulin optimisation, should this criteria (i.e. kidney) be taken into account since no 'intervention' is currently needed in terms of the patient's CKD? Or should the statement just state: Presence of altered organ function of at least one organ. |
| Expert C | Suggest: to remove .."such that...." as it is confusing.  Suggest: to replace with ' in which requiring drug dose monitoring and modification therapeutically'.....thus more relevant to pharmacist point of view |
| Expert D | Presence of altered organ function (at least one organ) in an acutely ill patient such that homeostasis cannot be maintained without pharmaceutical intervention |
| Expert E | Yes , definitions for organ dysfunctions , multiple organ failure will overscore points |
| Expert F | Yes. Suggest to clarify the extent of organ involvement which alter the PKPD of the medications in acute state Kidney: AKI, ESRF Brain: Low GCS? Traumatic brain injury? (Mild TBI doesn't need to be prioritized. Only severe TBI will alter PKPD of drug) Liver: Transaminitis, acute liver failure Lung: ? (Please clarify. Simple AEBA/ AECOPD case doesn't need extensive clerking) Bone marrow: ? (Please clarify. It may vary between the characteristic of wards. For example, bone marrow suppression is common for cancer and hematology patients) Heart: Decompensated heart failure |

**Question 3: (T) Therapeutic Drug Monitoring -** Medication(s) that has a narrow therapeutic range and requires individualisation of dosage regimen by maintaining a certain plasma or blood concentration within a targeted therapeutic window.

Experts’ responses

| **Likert Scale** | **Relevance** | **Completeness** |
| --- | --- | --- |
| 1 | 0 (0%) | 0 (0%) |
| 2 | 1 (4.2%) | 0 (0%) |
| 3 | 0 (0%) | 3 (12.5%) |
| 4 | 3 (12.5%) | 6 (25%) |
| 5 | 20 (83.3%) | 15 (62.5%) |

**Consensus

|  | % | **Consensus achieved** |
| --- | --- | --- |
| Relevance | 95.8 | Yes |
| Completeness | 87.5% | Yes |

**Consensus is achieved if ≥75% of experts rates agree or strongly agree in the Likert scale

1. *(a) Does the criteria above require any modification and addition in terms of content?*

Total number of comments/ suggestions: 4

Suggestions/comments given by the experts:

| Expert A | Does this include toxic cases? (e.g BDZ/ PCM/etc) |
| --- | --- |
| Expert B | Yes. Kindly consider to add toxicity and compliance assessment as the indications for TDM |
| Expert C | 1 TDM drug - 1 point? Or yes = 1 point? |
| Expert D | Yes, would focus on patient with tdm drugs that are inadequately responsive instead of all tdm drugs wherever applicable |

1. *(b) Does the criteria above require any modification in terms of sentence structure?*

Total number of comments/ suggestions: 6

Suggestions/comments given by the experts:

| Expert A | Does this include toxic cases? (e.g BDZ/ PCM/etc). |
| --- | --- |
| Expert B | Yes. |
| Expert C | Patient on medication(s) with narrow therapeutic index requiring Therapeutic Drug Monitoring (TDM). ** The purpose of TDM is self-explanatory; hence, it doesn't require explanation in the statement sentence. |
| Expert D | Suggest: To adjust the column/spacing to fit drug name written there. |
| Expert E | Medication(s) with narrow therapeutic ranges and require individualisation of dosage regimens by maintaining plasma or blood concentrations within targeted therapeutic windows. |
| Expert F | Medication with narrow therapeutic range and requires individualization of dosage regimen to maintain a certain plasma or blood concentration within a targeted therapeutic window to elicit a desired effect. |

**Question 4: (J) JKUT Quota Medication -** Patient requiring JKUT medication(s) HKL that has limited quota.

Experts’ responses

| **Likert Scale** | **Relevance** | **Completeness** |
| --- | --- | --- |
| 1 | 1 (4.2%) | 1 (4.2%) |
| 2 | 5 (20.8%) | 4 (16.7%) |
| 3 | 6 (25%) | 6 (25%) |
| 4 | 7 (29.2%) | 8 (33.3%) |
| 5 | 5 (20.8%) | 5 (20.8%) |

**Consensus

|  | % | **Consensus achieved** |
| --- | --- | --- |
| Relevance | 50 | No |
| Completeness | 54.1 | No |

**Consensus is achieved if ≥75% of experts rates agree or strongly agree in the Likert scale

1. *(a) Does the criteria above require any modification and addition in terms of content?*

Total number of comments/ suggestions: 9

Suggestions/comments given by the experts:

| Expert A | Add the updated [date] of JKUT |
| --- | --- |
| Expert B | 1 JKUT (with quota) = 1 point? Or yes = 1 point? |
| Expert C | The purpose of adding in the entire list of JKUT should be re-evaluated. If it is in terms of cost and the need to justify this cost expenditure on the patient then perhaps the list can be narrowed down to high-costing drugs that will make significant impact in terms of budgeting. (Also, Tinzaparin is no longer marketed in Malaysia as of 2019). |
| Expert D | Yes |
| Expert E | Suggest to include KPK item |
| Expert F | JKUT medication has quota could be due to high drug cost, not necessary need close monitoring from pharmacy in term of clinical outcome. |
| Expert G | I think this category serves a rather low priority as the determination of it being in the JKUT list is confounded largely by the cost. |
| Expert H | JKUT medication require pharmacist's additional attention to ensure continuation of medication supply. However, it is not a criteria for clinical pharmacy clerking. High alert medications are more relevant than JKUT. |
| Expert I | Suggest to add UKK (previously known as KPK) drugs too |

1. *(b) Does the criteria above require any modification in terms of sentence structure?*

Total number of comments/ suggestions: 9

Suggestions/comments given by the experts:

| Expert A | Yes |
| --- | --- |
| Expert B | Suggest to add in KPK drug |
| Expert C | Comment as mentioned above in 1.  The purpose of adding in the entire list of JKUT should be re-evaluated. If it is in terms of cost and the need to justify this cost expenditure on the patient then perhaps the list can be narrowed down to high-costing drugs that will make significant impact in terms of budgeting. (Also, Tinzaparin is no longer marketed in Malaysia as of 2019). |
| Expert D | Query: Why patient on JKTU drug need to prioritize for clerking? If patient bring JKTU POM? Any specific role on this context if patient plan to start with JKTU drugs. Suggestion: 'patient started with A* drugs that require monitoring / drug availability |
| Expert E | Suggest to include KPK item |
| Expert F | Suggest change to KPK drug instead of JKUT medications (because KPK not in FUKKM, which might need close monitoring by pharmacy for clinical outcome) |
| Expert G | Patient requiring HKL JKUT medication(s) with quotas |
| Expert H | Patient requiring JKUT medication(s) HKL due to unachievable treatment benefit and safety with conventional treatment |
| Expert I | JKUT (Controlled Medicine) |

**Question 5: (A) Anaesthetic Referral -** Disease state of a patient requiring ventilator support and referral to the anaesthetist.

Experts’ responses

| **Likert Scale** | **Relevance** | **Completeness** |
| --- | --- | --- |
| 1 | 0 (0%) | 0 (0%) |
| 2 | 3 (12.5%) | 2 (8.3%) |
| 3 | 1 (4.2%) | 4 (16.7%) |
| 4 | 6 (25%) | 7 (29.2%) |
| 5 | 14 (58.3%) | 11 (45.8%) |

**Consensus

|  | % | **Consensus achieved** |
| --- | --- | --- |
| Relevance | 83.3 | Yes |
| Completeness | 75 | Yes |

**Consensus is achieved if ≥75% of experts rates agree or strongly agree in the Likert scale

1. *(a) Does the criteria above require any modification and addition in terms of content?*

Total number of comments/ suggestions: 7

Suggestions/comments given by the experts:

| Expert A | Need to refine what you mean by ventilator support? Does non-invasive ventilator included? |
| --- | --- |
| Expert B | overlapping with organ dysfunction - lung + specialty care |
| Expert C | The relevance of this statement if questionable as patients who require ventilator support won't be warded in General Medical Wards. Even if they are initially admitted to the General Medical Wards, they will likely be transferred swiftly to the GICU for ventilator support. Other than that, the intention for anaesthetist referral is rather vague. Anaesthetist can be referred for pre-surgery evaluation; will this be considered as a criteria as well? |
| Expert D | Yes |
| Expert E | Intensivist, management of hemodynamic instability (eg shock, life threatening arrythmias) |
| Expert F | What about other forms of NIV which requires anaesthetic referrals such as BiPAP or CPAP? |
| Expert G | Yes. Suggest to change to "Anaesthetic referral/ Transfer from high dependency area". Besides patients who are referred to anaes for intubation, this apply to patients who are transferred/ discharged from ICU/ HDW also. |

1. *(b) Does the criteria above require any modification in terms of sentence structure?*

Total number of comments/ suggestions: 6

Suggestions/comments given by the experts:

| Expert A | Yes |
| --- | --- |
| Expert B | Under the circumstance as mentioned above, the statement could be altered to state: Patients with underlying conditions anticipated to require ventilator support. (e.g. in cases of impending respiratory collapse) |
| Expert C | Relevancy and is it can widely applied to general medical ward? Any specific monitoring by pharmacist if patient require ventilator support and anest referral? Pharmaceutical point of view? |
| Expert D | Is patient referred to anaesthetist for ventilation support/ Transferred out from intensive care? |
| Expert E | Disease state of a patient requiring ventilator support and anaesthetic referral |
| Expert F | As above.  Yes. Suggest to change to "Anaesthetic referral/ Transfer from high dependency area". Besides patients who are referred to anaes for intubation, this apply to patients who are transferred/ discharged from ICU/ HDW also. |

**Question 6: (M) Medication-related Admission -** Current admission is associated with drug related problems such as drug allergy and adverse drug events as well as toxic or poisoning cases.

Experts’ responses

| **Likert Scale** | **Relevance** | **Complete** |
| --- | --- | --- |
| 1 | 0 (0%) | 0 (0%) |
| 2 | 0 (0%) | 0 (0%) |
| 3 | 0 (0%) | 1 (4.2%) |
| 4 | 4 (16.7%) | 5 (20.8%) |
| 5 | 20 (83.3%) | 18 (75%) |

**Consensus

|  | % | **Consensus achieved** |
| --- | --- | --- |
| Relevance | 100 | Yes |
| Completeness | 95.8 | Yes |

**Consensus is achieved if ≥75% of experts rates agree or strongly agree in the Likert scale

1. *(a) Does the criteria above require any modification and addition in terms of content?*

Total number of comments/ suggestions: 4

Suggestions/comments given by the experts:

| Expert A | Suggest to include "drug related problems such as non-adherence" in the statement? |
| --- | --- |
| Expert B | Yes. Inclusive of occurrence of any drug-related problems (allergy, ADR etc) during the course of the admission. |
| Expert C | How about medication devices |
| Expert D | Drug related problem also includes underdose, overdose and medication error |

1. *(b) Does the criteria above require any modification in terms of sentence structure?*

Total number of comments/ suggestions: 1

Suggestions/comments given by the experts:

| Expert A | Yes. Unclear whether to include only those admissions related purely to any DRPs or may include those with DRPs occurring during the course of the ward stay. |
| --- | --- |

**Question 7: (S) Specialty Care -** Disease state of a patient requiring referral to other specialty care for their expert managements.

Experts’ responses

| **Likert Scale** | **Relevance** | **Complete** |
| --- | --- | --- |
| 1 | 0 (0%) | 0 (0%) |
| 2 | 0 (0%) | 2 (8.3%) |
| 3 | 3 (12.5%) | 3 (12.5%) |
| 4 | 5 (20.8%) | 4 (16.7%) |
| 5 | 16 (66.7%) | 15 (62.5%) |

**Consensus

|  | % | **Consensus achieved** |
| --- | --- | --- |
| Relevance | 87.5 | Yes |
| Completeness | 79.2 | Yes |

**Consensus is achieved if ≥75% of experts rates agree or strongly agree in the Likert scale

1. *(a) Does the criteria above require any modification and addition in terms of content?*

Total number of comments/ suggestions: 7

Suggestions/comments given by the experts:

| Expert A | To include Ryle Tube/ NG in the description. |
| --- | --- |
| Expert B | Suggest to include more specialties or leave as open statement |
| Expert C | Suggest add in patient with total parenteral nutrition and chemotherapy which need pharmacist to adjust the regimen according to patient condition. |
| Expert D | Define the list of choices and definition for specialty care - how about cardio? nephro? onco? gastro? hepato? hemato? |
| Expert E | May consider geriatric with polypharmacy if you think its relevant to narrow the scope because now higher number of elderly admitted |
| Expert F | Unsure if we are able to refer to other specialities |
| Expert G | Patient's risk assessment should not be solely based on specialty care referral.  Suggest to change "specialty care referral" to "patient-specific factors" such as: 1. Age (Young vs geriatric) 2. Frailty (physical or cognitive issues or incapacities) 3. Length of stay  4. Frequent presenter (multiple admission) 5. Multiple co-morbid conditions (use of multiple medications) 6. Adherence 7. Pregnancy/ lactation etc. |

**Question 8:**

1. *If you think that the draft PAST ignores some essential aspects in prioritizing patient’s acuity level in general medical ward, please list out each item in detail.*

Total number of comments/ suggestions: 17

Suggestions/comments given by the experts:

| Expert A | As mentioned in previous section. |
| --- | --- |
| Expert B | 1. KPK drug 2. TPN (rarely happen, but if got, important to clerk) |
| Expert C | 1. Patients stepped down for ICU care and/or still under review by peripheral ICU team. 2. Patients requiring special drug devices e.g. MDI, DPI, transdermal patches, GTN tablets (pharmacist counselling is important to ensure the best outcome). If such cases are clerked, the attending pharmacist will know better how to approach the patient effectively |
| Expert D | Yes. To focus more on pharmaceutical point of view |
| Expert E | New disease condition/ rare case that never clerked before. |
| Expert F | Suggest to include patient who on special devices which need pharmacy counselling eg. insulin pen, inhaler etc and patient with poor compliance of medicine |
| Expert G | 1) Patients with polypharmacy (e.g. on more than 10 regular medicines) or those with complex medication regimens, especially those with medications from multiple disciplines; or does this come under Part S (Specialty Care)?  2) Patients with extremes of weight (frail/obese) 3) Patients with compliance/adherence issues |
| Expert H | KPK drugs monitoring |
| Expert I | 1. J: Patient's on other medications such as KPK items, off-label use, ARF antibiotics are missed out. Patients on such medications also requires closer monitoring/review by clinical pharmacists. 2. T: Other medications that require close/extra monitoring such as warfarin (INR, drug-drug interactions), rifampicin (LFT, drug interactions) etc are missed out. |
| Expert J | Kindly consider adding medication mishandling/mismanagement issues under the Medication-related Admission category |
| Expert K | Drug interactions |
| Expert L | How about patients with special drug administration considerations ie stoma, RT, TPN etc. Medication delivery is equally important and requires close monitoring. Changes in administration may impact treatment outcome. Where will these group of patients lie in the category in the tool list? |
| Expert M | Clinical Fragility Score (CFS) more than 3 |
| Expert N | Allergies Comorbidies  Frailty Length of stay Adherence Multiple admission  Reason for admission Pregnancy/ lactation |
| Expert O | Please consider adding patients who are transferred from ICU or HDW (Step down) |
| Expert P | May include ADR / Allergy history |
| Expert Q | suggest to add patient with multiple drug therapy; patient with medical devices eg: inhaler; insulin etc |

1. *Referring to the total scoring, do you agree with the scoring method? If you do not, please state your reason and suggestion.*

| **Agree/Disagree** | **No. of Experts** |
| --- | --- |
| Agree | 12 |
| Disagree | 2 |
| No mention | 10 |

Total number of comments/ suggestions: 9

Suggestions/comments given by the experts:

| Expert A | Suggest to include criteria prioritization in the score. For example: IF the criteria T and/or M is marked during the assessment, the case has to be clerked (even though PAL fall under level 1. (Cadangan je :p) |
| --- | --- |
| Expert B | Suggest certain component/case, example: 1. Toxicity case - red flag case- high priority to do full clerking  2. Over warfarinization with PCC use |
| Expert C | Perhaps it will be more helpful if the basis for the current scoring is explained (i.e. how were the scores decided) so that the following comments can be more helpful. |
| Expert D | 0-1/2 points of pharmacotherapy review. 3 points- quick clerking. 4 and above full clerking |
| Expert E | Agree with scoring method which is divided into 3 level. But not sure how to decide the range of score and is there any reference or journal for the scoring method? |
| Expert F | No, the scoring seems like it will bring back a lot of high scoring points in general. The scoring total should be increased |
| Expert G | How or to what reference do you refer to decide for cut off at 3 for low priority cases? |
| Expert H | Just to clarify, is the maximum score 7? |
| Expert I | 0-2: pharmacotherapy review;  3-5: quick review; 6 and above: full clerking |

**Delphi Survey Round 2 Results – Expert Feedback**

**Total number of experts: 23 (Response rate: 23/24 = 95.8%)**

**Question 1**

**High Alert Medications:** Patient on High Alert Medications (HAM) for more than 24 hours

|  | **No of experts voting** | | | | | N = no of expert voted 4 and 5 (%) | Consensus reached?  ( Yes if > 75% voted 4 or 5) |
| --- | --- | --- | --- | --- | --- | --- | --- |
| Likert scale scoring | 1 | 2 | 3 | 4 | 5 |  |  |
| **Relevance** | **0** | **0** | **1** | **11** | **11** | **22 (95.7%)** | **Yes** |
| **Completeness** | **0** | **1** | **2** | **8** | **12** | **20 (87%)** | **Yes** |

Expert commented (3)

**Suggestion / comments given by the experts:** -

1. Suggest to change sentence to "Please specify if applicable". Also unclear since in the heading it says "1 point for 1 issue" then at the bottom it says (1 point for 1 drug). At a glance it is not clear how to score this
2. Is there a reason to cap the maximum number of HAMs to 2? There may be situations in which this part of the PAST may have higher weightage compared to the other criteria in this tool. If the patient is on many HAMs, this criteria alone should constitute consideration for the need for case to be clerked (High priority) since our main role involves pharmacotherapy.
3. Is repeated STAT dosing included in the definition of more than 24 hours? For example, repeated dosing for electrolyte corrections or repeated dosing for digoxin or amiodarone, where the gap between 1 STAT doses to another is more than 24 hours.

**Question 2**

**Organ Dysfunction:** Patient with one OR more acutely decompensated organ requiring pharmaceutical intervention

|  | **No of experts voting** | | | | | N = no of expert voted 4 and 5 (%) | Consensus reached?  ( Yes if > 75% voted 4 or 5) |
| --- | --- | --- | --- | --- | --- | --- | --- |
| Likert scale scoring | 1 | 2 | 3 | 4 | 5 |  |  |
| **Relevance** |  |  |  |  |  |  |  |
| **Main Statement** | **0** | **0** | **3** | **3** | **17** | **20 (87%)** | **Yes** |
| Brain | 1 | 0 | 6 | 5 | 11 | 16 (69.6%) | No |
| Heart | 0 | 0 | 3 | 5 | 15 | 20 (87%) | Yes |
| Kidney | 0 | 0 | 1 | 4 | 18 | 22 (95.7%) | Yes |
| Liver | 0 | 0 | 1 | 4 | 18 | 22 (95.7%) | Yes |
| Lungs | 0 | 0 | 5 | 5 | 13 | 18 (78.3%) | Yes |
| Bone Marrow | 0 | 0 | 6 | 7 | 10 | 17 (73.9%) | No |
| **Completeness** | **0** | **1** | **3** | **5** | **14** | **19 (82.6%)** | **Yes** |

Expert commented (5)

**Suggestion / comments given by the experts:**

1. Hard to classify what is meant by brain dysfunction, unless what is meant is to identify a decline in brain functioning (eg. stroke, brain metastasis) leading to cognitive decline therefore there are certain medications we have to use with care/does it mean that we need to consider if the medication has to cross the blood brain barrier
2. Just to clarify, since it is for acute decompensated organ, do you mean that... AKI - 1 point, CKD or ESRF on dialysis - 0 point? Acute liver injury - 1 point, chronic liver failure - 0 point? I think chronic kidney disease, chronic liver failure require pharmaceutical intervention such as dosage adjustment
3. Unclear statement. How to define brain/bone marrow/heart dysfunction? Any level of severity to eligible for 1 point?
4. The phrase 'acute' may be stumbling and confusing unless we aim to only prioritize such patients with acutely decompensated organ function and exclude those with chronic organ dysfunction. E.g If a CKD patient is admitted for treatment of cellulitis, there may be confusion as whether to score 'kidney' for organ dysfunction or not since the underlying CKD is not acute but will definitely impact antibiotic therapy (PKPD). Do we exclude such patients in our priority scoring? If we wish to include them and see them as priority then perhaps the sentence can be rephrased as "Presence of at least one organ dysfunction requiring pharmaceutical intervention".
5. Suggest to include relevant organs that highly affect PKPD if the statement refers to requirement of pharmaceutical intervention (ex: dosing adjustment/pharmacotherapy changes) due to altered organ function/s. Plus, the max score allowed is only 2 despite of the presence of 'multiorgan' failure

**Question 3**

**TDM:** Patient on medication(s) with narrow therapeutic index requiring TDM

|  | **No of experts voting** | | | | | N = no of expert voted 4 and 5 (%) | Consensus reached?  ( Yes if > 75% voted 4 or 5) |
| --- | --- | --- | --- | --- | --- | --- | --- |
| Likert scale scoring | 1 | 2 | 3 | 4 | 5 |  |  |
| **Relevance** | **0** | **0** | **0** | **6** | **17** | **23 (100%)** | **Yes** |
| **Completeness** | **0** | **2** | **0** | **6** | **15** | **21 (91.3%)** | **Yes** |

Expert commented (6)

**Suggestion / comments given by the experts:**

1. Would “suspected toxicity case” be more accurate
2. Do you mean that suspected toxicity case for above medications will be scored extra 1 point? For example, PCM 1 point + PCM toxicity 1 point = 2 points? Valproate 1 point + suspected valproate toxicity 1 point = 2 point? If so, suggest to remove the toxicity case and put it in the description "Patient on medications with narrow therapeutic index require TDM for efficacy OR toxicity"
3. Is the scoring 1 point for 1 drug group? If so, is there a significance to group the drugs and hence limiting then scoring? E.g. if a patient is on 3 AEDs that require TDM but if it grouped to 1, then the score will end up being 1 only. However, in reality, TDM drugs will require pharmacist intervention and monitoring which in turn will require full clerking (it is important to consider all patient parameters including concurrent medications in order to make the best recommendation). What does 'toxicity case' refer to? This is rather vague. Perhaps it would be sufficient to just state under Therapeutic Drug Monitoring, Patient on medication(s) with narrow therapeutic index requiring Therapeutic Drug Monitoring (TDM) for toxicity and/or compliance assessment. Under the scoring it would be sufficient to state 1 point for each drug.
4. Suggest to add Digoxin in the drug selection.
5. Kindly removed "narrow therapeutic index" in view of not all listed drugs have narrow window
6. I think can consider remove PCM or salicylate because it included in toxicity cases only

**Question 4**

**Special Drugs for Monitoring**

|  | **No of experts voting** | | | | | N = no of expert voted 4 and 5 (%) | Consensus reached?  (Yes if > 75% voted 4 or 5) |
| --- | --- | --- | --- | --- | --- | --- | --- |
| Likert scale scoring | 1 | 2 | 3 | 4 | 5 |  |  |
| **Relevance** |  |  |  |  |  |  |  |
| **4. Special Drugs for Monitoring (main statement)** | **0** | **0** | **2** | **5** | **16** | **21 (91.3%)** | **Yes** |
| Antibiotics in ARF | 0 | 0 | 2 | 4 | 17 | 21 (91.3%) | Yes |
| Anti-TB | 0 | 0 | 3 | 5 | 15 | 20 (86.9%) | Yes |
| Biologics | 0 | 0 | 3 | 1 | 19 | 20 (86.9%) | Yes |
| Parenteral Iron | 0 | 1 | 3 | 6 | 13 | 19 (82.6%) | Yes |
| IVIG | 0 | 1 | 1 | 7 | 14 | 21 (91.3%) | Yes |
| PCC | 0 | 0 | 3 | 4 | 16 | 20 (86.9%) | Yes |
| UKK | 1 | 0 | 3 | 7 | 12 | 19 (82.6%) | Yes |
| **Completeness** | **0** | **1** | **2** | **8** | **12** | **20 (86.9%)** | **Yes** |

Expert commented (9)

**Suggestion / comments given by the experts:**

1. Define the need for close monitoring. If it is monitoring in terms of drug usage (related to cost) then it does not constitute priority in the need for case clerking. If it is the case for monitoring of side effects, drugs listed (e.g. Venofer/ Cosmofer/ IVIG), the effects if any, will be seen during the course of administration. If the drug has already been given and completed (uneventful) prior to the pharmacist evaluating the case, should this be also be scored? Even though the event (drug administration) has already been completed.
2. UKK referring to?
3. Suggest to add in chemotherapy/cancer treatment as oncology/hematology patients may be warded in other disciplines (eg. medical ward).
4. Special drugs definition? OR suggest to use other term e.g Specific drugs/ medicines/medication.
5. 1. Biologics: suggest to add Omalizumab 2. UKK drugs?
6. Anticoagulant?
7. UKK drugs- column to list down drug involved; to include JKUT drugs for close monitoring as well.
8. Antibiotics listed in ARF: Some of the antibiotics not listed in ARF are worth attention, especially those antibiotics using very high dose. For example, high dose Unasyn or Sulperazone for acinetobacter, high dose ceftazidime and cefepime.
9. The special drugs for close monitoring only inclusive of the ones mentioned above? or it is based on the types of cases we encounter, for example anti psychotics/mood stabilisers, transplant drugs and etc.

**Question 5**

**Intensive Care Transition:** Patient requiring referral to OR transition of care from critical Care Team

|  | **No of experts voting** | | | | | N = no of expert voted 4 and 5 (%) | Consensus reached?  ( Yes if > 75% voted 4 or 5) |
| --- | --- | --- | --- | --- | --- | --- | --- |
| Likert scale scoring | 1 | 2 | 3 | 4 | 5 |  |  |
| **Relevance** | **0** | **0** | **0** | **10** | **13** | **23 (100%)** | **Yes** |
| **Completeness** | **0** | **1** | **1** | **8** | **13** | **21 (91.3%)** | **Yes** |

Expert commented (2)

**Suggestion / comments given by the experts:**

1. Suggest to change the statement to "Reason for referral or transition of care".
2. Can we add: '.....that require pharmacotherapy adjustment/pharmaceutical intervention'.

**Question 6**

**Medication Related Issue:** Drug related issues leading to hospitalization OR identified during ward stay

|  | **No of experts voting** | | | | | N = no of expert voted 4 and 5 (%) | Consensus reached?  ( Yes if > 75% voted 4 or 5) |
| --- | --- | --- | --- | --- | --- | --- | --- |
| Likert scale scoring | 1 | 2 | 3 | 4 | 5 |  |  |
| **Relevance** |  |  |  |  |  |  |  |
| **Medication related issue (main statement)** | **0** | **0** | **1** | **4** | **18** | **22 (95.6%)** | **Yes** |
| Administration | 0 | 0 | 4 | 5 | 14 | 19 (82.6%) | Yes |
| ADR | 0 | 0 | 1 | 2 | 20 | 22 (95.6%) | Yes |
| Device related | 0 | 1 | 2 | 10 | 10 | 20 (86.9%) | Yes |
| Drug interactions | 0 | 1 | 0 | 5 | 17 | 22 (95.6%) | Yes |
| Medication error | 0 | 0 | 2 | 6 | 15 | 21 (91.3%) | Yes |
| Non-adherence | 0 | 0 | 1 | 10 | 12 | 22 (95.6%) | Yes |
| Polypharmacy | 0 | 0 | 0 | 6 | 17 | 23 (100%) | Yes |
| Underdose/overdose | 0 | 0 | 0 | 4 | 19 | 23 (100%) | Yes |
| **Completeness** | **0** | **1** | **2** | **5** | **15** | **20 (86.9%)** | **Yes** |

Expert commented (5)

**Suggestion / comments given by the experts:**

1. Device-related meaning? - Insulin pen spoil, so not inject insulin at home??
2. Suggest to change statement to "Nasogastric tube" instead of Ryle's tube.
3. Suggest to allocate max 3 points for this section.
4. Suggest to add Poisoning case where pharmacist expert on antidote usage are required.
5. How is administration (e.g. Ryles Tube) different from Device-related?

**Question 7:**

**Specialty Care Referral:** *-* Patient under certain specialty care for their expert management requiring pharmaceutical monitoring

|  | **No of experts voting** | | | | | N = no of expert voted 4 and 5 (%) | Consensus reached?  ( Yes if > 75% voted 4 or 5) |
| --- | --- | --- | --- | --- | --- | --- | --- |
| Likert scale scoring | 1 | 2 | 3 | 4 | 5 |  |  |
| **Relevance** |  |  |  |  |  |  |  |
| **Specialty Care Referral (Main Statement)** | **0** | **0** | **6** | **6** | **11** | **17 (86.9%)** | **Yes** |
| Gastroenterology | 0 | 0 | 6 | 6 | 11 | 17 (73.9%) | No |
| Geriatric | 0 | 0 | 3 | 6 | 14 | 20 (87%) | Yes |
| Hematology | 0 | 0 | 3 | 7 | 13 | 20 (86.9%) | Yes |
| Infectious Disease | 0 | 0 | 2 | 6 | 15 | 21 (91.3%) | Yes |
| Palliative care/APS | 0 | 0 | 3 | 8 | 12 | 20 (87%) | Yes |
| Rheumatology | 0 | 0 | 4 | 5 | 14 | 19 (82.6%) | Yes |
| **Completeness** | **1** | **2** | **0** | **9** | **11** | **20 (86.9%)** | **Yes** |

Expert commented (5)

**Suggestion / comments given by the experts:**

1. Is there a need to limit the specialty referrals to only certain disciplines as listed? Perhaps it would be more appropriate to leave this statement open-ended. Scoring can be 1 point for each specialty referral.
2. Suggest to add: Respiratory including TB, ASTHMA, COPD (referral case), Cancer patient on Chemotherapy or targeted therapy
3. Agree if patient with special care and need close monitoring. However, in HKL, eg geriatric ward, the clinical pharmacist need to select based on patient's condition (not necessary clerk 100% geriatric patient in ward)
4. I think there are some overlapping with previous domain. For example, for those referred to APS/ palliative will definitely on strong opioid, so this will get (APS -1 point, narcotics -1 points) Refer to ID for carbapenem, colistin - 2 points in total (ID-1 point, ARF -1 point) That is why i think this domain is not complete, and there are some referrals are not in the list such as endocrine
5. how about other sub specialty care such as respiratory, psychiatry, endocrine and etc

**Question 8:**

**Patient Related Factor:**

|  | **No of experts voting** | | | | | N = no of expert voted 4 and 5 (%) | Consensus reached?  ( Yes if > 75% voted 4 or 5) |
| --- | --- | --- | --- | --- | --- | --- | --- |
| Likert scale scoring | 1 | 2 | 3 | 4 | 5 |  |  |
| **Relevance** |  |  |  |  |  |  |  |
| **Patient related factor (Main Statement)** | **0** | **0** | **2** | **4** | **17** | **21 (91.3%)** | **Yes** |
| History of ADR/Allergy | 0 | 0 | 2 | 6 | 15 | 21 (91.3%) | Yes |
| History of Fall | 0 | 1 | 4 | 7 | 11 | 18 (78.2%) | Yes |
| Multiple admissions | 0 | 0 | 3 | 4 | 16 | 20 (87%) | Yes |
| Special populations | 0 | 0 | 3 | 5 | 15 | 20 (86.9%) | Yes |
| **Completeness** | **0** | **1** | **1** | **7** | **14** | **21 (91.3%)** | **Yes** |

Expert commented (3)

**Suggestion / comments given by the experts:**

1. Suggest to put Non-adherence under this section instead of medication-related. Suggest reduce max point to 1 instead of 2.
2. Other special population to be considered: Underweight / cachexia, immunosuppressed patient This journal gives some idea of special populations/ patient related factor which can be a reference (Table 2 and Table 3): <https://journal.chestnet.org/article/S0012-3692(15)51991-0/fulltext>
3. How about those immunocompromised? Do we include them in the special population category?

**Question 9:**

**If you think that the draft PAST ignores some essential aspects in prioritizing patient acuity in GENERAL MEDICAL ward, please list out each item in detail.**

Experts commented: 6

**Suggestion / comments given by the experts:**

1. Antifungal use

2. Dengue fever, urology and gynae related issues

3. It Cover all essential aspects

4. N/A

5. NIL

6. No

**10a.**

**Referring to the total scoring, do you agree with the scoring method and its corresponding clerking priority?**

| No of experts agreed | 20 (87%) |
| --- | --- |
| No of experts disagreed | 3 (13%) |

**10b.**

**If you do not agree with the scoring method and its corresponding clerking priority, please state your reasons and suggestions in detail.**

Experts commented: 3

**Suggestion / comments given by the experts:**

1. Perhaps a maximum point shouldn't be required per criteria as this will limit the scoring in essential criteria (E.g. HAM).
   If would be helpful to understand the basis for the current suggested scoring in order to give feedback on this.
2. More details are needed to individualised treatment to your specialty, the scoring doesn’t not dictate the relevance of priority, it would still come down to clinical judgement which also varies from day to day, thank you
3. Some of the scoring is overlapping as i mentioned in previous section.
   The prescribing pattern of the drug (HAM, special drug) is strongly correlate with the specialty referral.
   For example, APS referral 1 point, narcotics 1 point = points. Same to ID with antibiotics, Rheumatology with biologics and steroids.
   If this is your intention to score this kind of patient with higher marks, then just ignore this comment. If not, the specialty referral part is not suitable

**Delphi Survey Round 3 Results – Expert Feedback**

**Total number of experts responded: 24 (Response rate: 24/24 = 100%)**

**Question 1**

**High Alert Medications:** Patient on High Alert Medications (HAM) for more than 24 hours

No rating done for Round 3.

Expert commented (6)

**Suggestion / comments given by the experts:**

1. None
2. N/a
3. No comment
4. Relevant and complete
5. Ok
6. Agree

|  | **No of experts voting** | | | | | N = no of expert voted 4 and 5 (%) | Consensus reached?  ( Yes if > 75% voted 4 or 5) |
| --- | --- | --- | --- | --- | --- | --- | --- |
| Likert scale scoring | 1 | 2 | 3 | 4 | 5 |  |  |
| **Relevance** |  |  |  |  |  |  |  |
| **Main statement** | **0** | **0** | **1** | **5** | **18** | **23 (95.8%)** | **Yes** |
| Heart | 0 | 0 | 2 | 5 | 17 | 22 (91.6%) | Yes |
| Kidney | 0 | 0 | 1 | 3 | 20 | 23 (95.8%) | Yes |
| Liver | 0 | 0 | 1 | 6 | 17 | 23 (95.8%) | Yes |
| Lungs | 0 | 0 | 4 | 5 | 15 | 20 (83.3%) | Yes |
| **Completeness** | **0** | **0** | **1** | **7** | **16** | **21 (95.8%)** | **Yes** |

**Question 2**

**Organ Dysfunction:** Patient with one OR more decompensated organ requiring pharmaceutical intervention

Expert commented (4)

**Suggestion / comments given by the experts:**

1. Suggest to amend the statement to: Patient with one OR more decompensated organ function(s) requiring pharmaceutical intervention (V)
2. Suggest to use more scientific language for liver cancer eg. liver carcinoma/liver neoplasm (E)
3. What about CNS? Patients with ischemic/hemorrhagic stroke , TBI, seizures esp refractory (C)
4. Agree

**Question 3**

**Therapeutic Drug Monitoring:** Patient on medication(s) that requires Therapeutic Drug Monitoring (TDM)

- Refer Appendix 2 – TDM list

|  | **No of experts voting** | | | | | N = no of expert voted 4 and 5 (%) | Consensus reached?  ( Yes if > 75% voted 4 or 5) |
| --- | --- | --- | --- | --- | --- | --- | --- |
| Likert scale scoring | 1 | 2 | 3 | 4 | 5 |  |  |
| **Relevance** | **0** | **1** | **0** | **4** | **19** | **23 (95.8%)** | **Yes** |
| **Completeness** | **0** | **1** | **0** | **5** | **18** | **23 (95.8%)** | **Yes** |

Expert commented (3)

**Suggestion / comments given by the experts:**

1. Suggest to rephrase as "Add 1 point for *a* suspected toxic case"
2. Does all TDM cases need to be clerked? Maybe we can help provide a set of criteria on which cases require definite clerking? just an idea
3. Agree

**Question 4:**

**Specific Drugs for Close Monitoring**

Drugs that require close monitoring for their:

1. Side effects/adverse events
2. Infusion related reaction
3. Therapeutic efficacy

|  | **No of experts voting** | | | | | N = no of expert voted 4 and 5 (%) | Consensus reached?  ( Yes if > 75% voted 4 or 5) |
| --- | --- | --- | --- | --- | --- | --- | --- |
| Likert scale scoring | 1 | 2 | 3 | 4 | 5 |  |  |
| **Relevance** |  |  |  |  |  |  |  |
| **4. Special drug for close monitoring (Main statement)** | **0** | **0** | **0** | **7** | **17** | **24 (100%)** | **Yes** |
| Antibiotics in ARF | 0 | 0 | 2 | 3 | 19 | 22 (91.6%) | Yes |
| Anti-TB | 0 | 0 | 2 | 4 | 18 | 22 (91.6%) | Yes |
| Biologics | 0 | 0 | 1 | 3 | 20 | 23 (95.8%) | Yes |
| IV Iron | 0 | 1 | 0 | 5 | 18 | 23 (95.8%) | Yes |
| IVIG | 0 | 0 | 2 | 6 | 16 | 22 (91.6%) | Yes |
| PCC | 0 | 0 | 2 | 4 | 18 | 22 (91.6%) | Yes |
| UKK | 0 | 0 | 4 | 9 | 11 | 20 (83.3%) | Yes |
| **Completeness** | **0** | **0** | **3** | **5** | **16** | **21 (87.5%)** | **Yes** |

Expert commented (6)

**Suggestion / comments given by the experts:**

1. Suggest to rephrase as "infusion-related reaction
2. Suggest to change 'infusion related reaction' to 'administration' which includes dilution/infusion/ recon etc.
3. All Abbreviations for all short forms used should be explained in appendix. Reason 1): PRP or even PF might not knowing all the abbreviations stated and + Easier for future data analysis or research.- (in past tool no abbreviation used)
4. UKK?
5. Suggest to put in Antimicrobial Request Form (ARF), Ubat Kelulusan Khas (UKK), Adverse Drug Reaction (ADR)
6. To add strong opioids

**Question 5. Intensive / Critical Care Transition**

Patient requiring referral to OR transition of care from critical care team

No rating done in Delphi Round 3.

Expert commented (1)

**Suggestion / comment given by the experts:**

1. Agree

**Question 6:**

**Medications-Related Issues**

Drug related issues leading to hospitalization OR identified during ward stay

|  | **No of experts voting** | | | | | N = no of expert voted 4 and 5 (%) | Consensus reached?  ( Yes if > 75% voted 4 or 5) |
| --- | --- | --- | --- | --- | --- | --- | --- |
| Likert scale scoring | 1 | 2 | 3 | 4 | 5 |  |  |
| **Relevance** |  |  |  |  |  |  |  |
| **6. Medications Related Issues (Main statement)** | **0** | **0** | **2** | **6** | **16** | **22 (91.6%)** | **Yes** |
| Administration | 0 | 0 | 3 | 6 | 15 | 21 (87.5%) | Yes |
| ADR | 0 | 0 | 1 | 5 | 18 | 23 (95.8%) | Yes |
| Device related | 0 | 0 | 1 | 10 | 13 | 23 (95.8%) | Yes |
| Drug-drug interaction | 0 | 0 | 2 | 5 | 17 | 22 (91.6%) | Yes |
| Medication errors | 0 | 1 | 0 | 4 | 19 | 23 (95.8%) | Yes |
| Non-adherence | 0 | 0 | 2 | 6 | 16 | 22 (91.6%) | Yes |
| Polypharmacy | 0 | 0 | 2 | 6 | 16 | 22 (91.6%) | Yes |
| Overdose/underdose | 0 | 0 | 2 | 5 | 17 | 22 (91.6%) | Yes |
| **Completeness** | **0** | **0** | **1** | **8** | **15** | 23 (95.8%) | **Yes** |

Expert commented (4)

**Suggestion / comments given by the experts:**

1. Suggest to amend statement to: Medication-related Issues
2. How about patient related ie improper/incorrect use device etc
3. Unclear statement about device-related
4. Poly pharmacy: kindly state more than 5 drugs

**Question 7:**

**Specialty Care Referral:** *-* Patient under certain specialty care for their expert management requiring pharmaceutical monitoring

|  | **No of experts voting** | | | | | N = no of expert voted 4 and 5 (%) | Consensus reached?  ( Yes if > 75% voted 4 or 5) |
| --- | --- | --- | --- | --- | --- | --- | --- |
| Likert scale scoring | 1 | 2 | 3 | 4 | 5 |  |  |
| **Relevance** |  |  |  |  |  |  |  |
| Specialty Care Referral (Main Statement) | 1 | 1 | 1 | 7 | 14 | 21 (87.5%) | Yes |
| Geriatric | 1 | 0 | 2 | 7 | 14 | 21 (87.5%) | Yes |
| Hematology | 1 | 0 | 3 | 5 | 15 | 20 (83.3%) | Yes |
| Infectious Disease | 1 | 0 | 2 | 5 | 16 | 21 (87.5%) | Yes |
| Palliative care/APS | 0 | 0 | 1 | 8 | 15 | 23 (95.8%) | Yes |
| Rheumatology | 1 | 1 | 2 | 5 | 15 | 20 (83.3%) | Yes |
| **Completeness** | **1** | **1** | **2** | **5** | **15** | **20 (83.3%)** | **Yes** |

Expert commented (6)

**Suggestion / comments given by the experts:**

1. Strongly agree if for general ward. However, in HKL with some wards (eg. Geriatric ward), the selection of patients to be prioritized for provision of pharmaceutical care could be based on other criteria
2. In view that our main concern as clinical pharmacists is to do with drug-related issues, then this session is actually already represented in section S (Specific Drugs for Close Monitoring) since there is an “Others” option can that be filled if the list is not exhaustive. Our concern should be what drugs the patient is started by being referred to these subspecialties (ie started by these subspecialities) and not by the fact of the referral
3. Does Palliative Care have to be shared with APS? Some patients are referred to Palliative Care for symptoms other than pain or for the initiation of palliative care
4. Suggest to rephrase as “Patients who are under specialty care for their expert management and requires pharmaceutical monitoring”
5. The percentage of cases with Cardio & AKI/CKD/ESRF in medical ward surpasses some components listed here. These disciplines should be given more consideration
6. Suggest to remove this section as it is redundant.
   1. Expert management requiring pharmaceutical monitoring is already given points in other sections, such as:
   2. Infectious disease: already given points for ARF
   3. Haematology – Venofer, PCC, etc.

**Question 8:**

**Patient related factor:**

|  | **No of experts voting** | | | | | N = no of expert voted 4 and 5 (%) | Consensus reached?  ( Yes if > 75% voted 4 or 5) |
| --- | --- | --- | --- | --- | --- | --- | --- |
| Likert scale scoring | 1 | 2 | 3 | 4 | 5 |  |  |
| **Relevance** |  |  |  |  |  |  |  |
| Patient related factor (Main Statement) | 0 | 0 | 4 | 5 | 15 | 20 (83.3%) | Yes |
| History of ADR/Allergy | 0 | 0 | 3 | 4 | 17 | 21 (87.5%) | Yes |
| History of Fall | 0 | 1 | 7 | 1 | 15 | 16 (66.7%) | No |
| Multiple admissions | 0 | 1 | 2 | 6 | 15 | 21 (87.5%) | Yes |
| Special populations – Obesity/Underweight | 0 | 0 | 3 | 7 | 14 | 21 (87.5%) | Yes |
| Special populations – Pregnancy/Lactation | 0 | 0 | 5 | 3 | 16 | 19 (79.2%) | Yes |
| Special populations – Immunocompromised | 0 | 0 | 1 | 7 | 16 | 23 (95.9%) | Yes |
| **Completeness** | **0** | **0** | **4** | **5** | **15** | **20 (83.3%)** | **Yes** |

Expert commented (3)

**Suggestion / comments given by the experts:**

1. Relevance of “history of fall” – if it is drug-related then yes. Relevance of “multiple admissions” – if it is drug-related (either to do with drug adherence etc) then yes, it will be relevant
2. For those with history of fall, maybe can put a note: Fall due to drug (FRIDs) as not all falls are related to drug use. Some may had fall at home or in the ward unrelated to drug/medical condition
3. Suggest to move the geriatric referral in the section to special populations geriatrics to section P

**Question 9:**

**If you think that the draft PAST ignores some essential aspects in prioritizing patient acuity in GENERAL MEDICAL ward, please list out each item in detail.**

Expert commented (3)

**Suggestion / comments given by the experts:**

1. N/A
2. No
3. Nil

**10a. Referring to the total scoring, do you agree with the scoring method and its corresponding clerking priority?**

| No of experts agreed | 23 (95.8%) |
| --- | --- |
| No of experts disagreed | 1 (4.2%) |

**10b. If you do not agree with the scoring method and its corresponding clerking priority, please state your reasons and suggestions in detail.**

Expert commented (1)

**Suggestion / comments given by the experts:**

1. Clerking is individualised, as a whole with magnitude of reasons
