# Supplementary material for: Development and validation of a pharmaceutical assessment screening tool to prioritise patient care in a tertiary care hospital
Source: PLoS One. 2023 Mar 3;18(3):e0282342. doi: 10.1371/journal.pone.0282342 (PMC9983860; doi:10.1371/journal.pone.0282342)
Supplement: Attachment — Submitted filename: Response to Reviewers.pdf [file pone.0282342.s003.pdf]

Journal Submission Rebuttal Letter

Emily Chenette
Editor-in-Chief
PLOS-ONE

15th December 2022

Dear Professor Emily,

Thank you for inviting us to submit a revised draft of our entitled ‘Development and validation of a pharmaceutical assessment screening tool to prioritise patient care in a tertiary care hospital’ for publication as a research article in the *PLOS ONE* journal. We also appreciate the time and effort you and each reviewer have dedicated to providing insightful opinions on ways to strengthen our paper. Thus, it is with great pleasure that we resubmit our article for further consideration. We have incorporated changes that reflect the detailed suggestions you have graciously provided.

To facilitate your review of our revisions, we attached a document for the point-by-point response to the questions and comments delivered in your email dated 29 November 2022.

Again, thank you for allowing us to strengthen our manuscript with your valuable comments and queries. We look forward to hearing from you regarding our submission. We would be glad to respond to any further questions and comments that you may have.

We hope that the revisions in the manuscript and our accompanying response will be sufficient to make our manuscript suitable for publication in the *PLOS ONE* journal.

Thank you for your time and attention.

Best wishes,

Cheok Ee Chang
Clinical pharmacist
Department of Pharmacy
Hospital Kuala Lumpur, Malaysia
Tel: +603-3181182
Carolce8@gmail.com

Responses to Reviewers' Comments

Journal : PLOS ONE
Submission ID : PONE-D-22-28558
Title : Development and validation of a pharmaceutical assessment screening tool to prioritise patient care in a tertiary care hospital

Authors : Cheok Ee Chang, Rahela Ambaras Khan, Chan Yen Tay, Baavaanii Thangaiyah, Victor Sheng Teck Ong, Sabariah Pakeer Oothuman, Shazwani Zulkifli, Nur Fatin Najwa Azemi, Pavithira Subramaniam

We thank the reviewers for thoroughly reading our manuscript and providing helpful comments and suggestions. We have addressed all issues indicated in the review report and hope that our revision has improved the paper to a level of an acceptable standard.

**Academic Editor**		
**No.**	**Comments**	**Responses**
1.	Please ensure that your manuscript meets PLOS ONE's style requirements, including those for file naming	The manuscript has been revised as per PLOS ONE's style requirements
2	Please provide additional details regarding participant consent. In the ethics statement in the Methods and online submission information, please ensure that you have specified (1) whether consent was informed and (2) what type you obtained (for instance, written or verbal, and if verbal, how it was documented and witnessed).	**Ethics Statement:**<br><br>Written informed consent has been obtained from all participating experts prior to conducting Delphi methods
3	In your Data Availability statement, you have not specified where the minimal data set underlying the results described in your manuscript can be found. PLOS defines a study's minimal data set as the underlying data used to reach the conclusions drawn in the manuscript and any additional data required to replicate the reported study findings in their entirety. All PLOS journals require that the minimal data set be made fully available	**Data availability:**<br><br>The minimum data set can be obtained via email to pastresearch.hkl@gmail.com

**Reviewer #1**		
**No.**	**Comments**	**Responses**
	The study "Development and validation of a pharmaceutical assessment screening tool to prioritise patient care in a tertiary care hospital" is well-conducted and reported. I have a few minor comments, which I think will make the reporting slightly better.<br><br>Minor:	We are grateful for the reviewer's kind comments.
1.	I could not find how the Patient acuity level scoring was performed. What weights were given to each criterion and/or sub-component? I am not sure if it is 1/0 scoring or some other weights for the 8 criteria, unless I overlooked it. I would recommend authors to add a sentence or two describing the scoring method that reached the consensus.	The patient acuity level (PAL) was first set with at least a total score of 3 and above to indicate a high acuity level (Line 116 – 117) but it was commented during Delphi Survey 1 where the Panel suggest stratifying patient into Level 1 and Level 2 and to increase the total threshold score for level 2 to be more than 'score 4 and above (Line 228 – 233). The patient acuity level and scoring were then modified to:<br>- Level 0: total score '0-1'<br>- Level 1: total score '2-6'<br>- Level 2: 7 and above respectively<br><br>The amended acuity level and scoring was accepted in Delphi Rounds 2 and 3 without any further amendment.<br><br>The information about the amended score and acuity level is summarised in **Table 5: Modified scoring system, PAL, and clerking priority in the PAST tool from Delphi round 2 and 3.**
2.	Were the experts involved in the pilot Delphi round also part of the Delphi surveys 1–3? I would recommend the authors to add a sentence	These 5 experts involved in the pilot Delphi round were not included in Delphi Survey Round 1-3. This information has been added as follows:

Reviewer #1		
**No.**	**Comments**	**Responses**
	regarding this in the methods.	These 5 experts were not included in Delphi Survey Round 1- 3.<br>**[Method; Validation of Past: Line 137 – 138]**
3	A typo: in table 2 first row, "High Alert Medication" is written twice.	We appreciate the reviewer's feedback in this matter. Amendment has been made in removing duplication words.<br><br>**[Results; Table 2: Line 216]**

Reviewer #2		
**No.**	**Comments**	**Responses**
1.	The manuscript presented an interesting introduction to the purpose of the study. However, the tools researchers developed for prioritizing patients for intensive pharmaceutical care is not new and has mostly adapted from published literature.	We are grateful for the reviewer's kind comments<br><br>We understand the concern of the reviewer on available published tools for prioritising patient care, however, the different settings might have different concerns or limitations in prioritising patient care.<br><br>Hence, our study has developed and validated a screening tool, STORIMAP which suits our local setting and may act as a comprehensive tool for clinical pharmacists to prioritise and optimise patient care in high-burden medical wards. We also incorporated a few criteria/domains such as 'High Alert Medications' and 'JKUT (Drug Review & Therapeutic Committee) Quota medication' that maybe differed from other available published tools. However, JKUT Quota medication has been removed after the Round 1 Delphi Survey.

Reviewer #2		
**No.**	**Comments**	**Responses**
2.	Most of the expert panel members were female (83.3%), Is it similar to the proportion of clinical pharmacist in the three selected hospitals to be a representative data?	Yes. 84.6 % of clinical pharmacists in these three hospitals are female (8 male and 44 female). Hence the input from the expert panels (majority female) can be representative data.
3.	Provide expansion of abbreviations used in table 2 in table legend.	Amendment has been made with the inclusion of table legend as follows:<br>- JKUT Drug Review & Therapeutic Committee<br><br>**[Results: Table 2: Line 216]**
4.	Again, all abbreviations are not expanded in table 3 legend.	Amendment has been made with the inclusion of table legend as follows:<br>- ARF: Antibiotic Request Form<br>- UKK: Ubat Kelulusan Khas<br>- ADR: Adverse Drug Reaction<br><br>**[Results: Table 3: Line 259]**
5.	The tool developed as PAST is presented merely in percentage and grading for consensus, how reliable this tool in the absence of reliability testing is not clear to me.	Delphi Method was chosen for developing PAST as it is an ideal platform where the expression and exchange of expert opinion can be quantified and measured. Through a series of rounds, Delphi Method is a reliable indicator for consensus.
6.	The sample size is too small to come to the conclusion of the reliability of the outcome	We understand the reviewer's concern about the low sample size of this research.<br><br>For reviewer information, even though the sample size is small, it basically represents universal sampling. We included all the experts in that three hospitals (HKL, HTA and IPR) that suits our inclusion criteria.
7.	I hope all participants who are said to have	Yes, years of experience in a clinical pharmacy unit

Reviewer #2		
**No.**	**Comments**	**Responses**
	experience in clinical pharmacy unit shall also by default will have experience of working in pharmacy as clinical pharmacy unit is within pharmacy. However, the demographics is confusing in table 1. I can find different levels of experience shown in this two categories. 9-10 years' experience with only 6 participants while there is no 9-10 years' data in clinical pharmacy, it jumps from 8-9 and then more than or equal to 10 years, there is no data of 9-10 years.	by default will have experience working in the pharmacy.<br><br>As per study inclusion criteria, the demographics presented in table 1 represent experts with a minimum of 7 years of working experience in the pharmacy field and with at least a Masters in clinical pharmacy.<br><br>The result shows that the working experience of our experts ranges from 9 to ≥14 years while their experience as clinical pharmacists ranges between ≤5 years to ≥10 years.
8.	What is distribution of participants from different centres to understand the diversification of them?	The distribution of institutions includes:<br>- HKL (General Hospital without paediatric/ obstetrics & gynaecology services; 1887 beds) = **16 experts recruited;**<br>- HTA (women and child hospital; 600 beds) = **7 experts recruited;**<br>- IPR (Respiratory Institute that manages respiratory/tuberculosis cases; 118 beds) = **1 expert recruited.**<br>All these hospitals are basically under one management. The staffs are rotated among these hospitals. General medical wards for adults in patients are available at HKL.
9.	The first three paragraphs of discussion are more less repeat of introduction; it needs to discuss the results with interpretation in consideration with other published work.	We take note of the reviewer's comment on this. The first three paragraphs have been revised and simplified.<br>[Discussion: Line 316 – Line 331]

Reviewer #2		
**No.**	**Comments**	**Responses**
		We cited more references to compare our results with other published works in terms of prioritisation based on antimicrobials and expensive medicines.<br><br>[Discussions: Line 349 And Line 380-381]
10.	How they ensured anonymity of the participants in this study especially if was done face to face, I hope at least a part of it was done face to face.	This Delphi Survey is conducted **through online Google Forms**.
11.	How this tool which author refer as STORIMAP is considered as validated? Authors claim in line 409.	STORIMAP which refers to the mnemonic of PAST is validated through three rounds of the Delphi Survey conducted.
12.	Do they have criteria, content and construct validity checked? Did they do reliability check?	The Delphi Survey method is based upon the assumption of safety in numbers in which several people are less likely to arrive at a wrong decision as compared to a single individual. Decisions are further strengthened by reasoned arguments in which assumptions are challenged, thus helping to enhance validity. Further, repeated rounds of the survey help to increase the concurrent validity (Hassan et al. Research guidelines for the Delphi survey technique. Journal of Advanced Nursing, 2000; 32(4), 1008 - 1015)
