# Supplementary material for: Development and validation of a pharmaceutical assessment screening tool to prioritise patient care in a tertiary care hospital
Source: PLoS One. 2023 Mar 3;18(3):e0282342. doi: 10.1371/journal.pone.0282342 (PMC9983860; doi:10.1371/journal.pone.0282342)
Supplement: Attachment — Submitted filename: PONE-D-22-28558_review1.docx [file pone.0282342.s002.docx]

The study “Development and validation of a pharmaceutical assessment screening tool to prioritise patient care in a tertiary care hospital” is well-conducted and reported. I have a few minor comments, which I think will make the reporting slightly better.

1. I could not find how the Patient acuity level scoring was performed. What weights were given to each criterion and/or sub-component. I am not sure if it is 1/0 scoring or some other weights for the 8 criteria, unless I overlooked it. I would recommend authors to add a sentence or two describing the scoring method that reached the consensus.
2. Were the experts involved in the pilot Delphi round also part of the Delphi surveys 1–3? I would recommend the authors to add a sentence regarding this in the methods.
3. A typo: in table 2 first row, “High Alert Medication” is written twice.
